# Global Emergence and Dissemination of Enterococci as Nosocomial Pathogens: Attack of the Clones?

**DOI:** 10.3389/fmicb.2016.00788

**Published:** 2016-05-26

**Authors:** Ana M. Guzman Prieto, Willem van Schaik, Malbert R. C. Rogers, Teresa M. Coque, Fernando Baquero, Jukka Corander, Rob J. L. Willems

**Affiliations:** ^1^Department of Medical Microbiology, University Medical Center UtrechtUtrecht, Netherlands; ^2^Hospital Universitario Ramon y Cajal, Instituto Ramón y Cajal de Investigación SanitariaMadrid, Spain; ^3^CIBER Epidemiología y Salud PúblicaMadrid, Spain; ^4^Unidad de Resistencia a Antibióticos y Virulencia Bacteriana Asociada al Consejo Superior de Investigaciones CientíficasMadrid, Spain; ^5^Department of Mathematics and Statistics, University of HelsinkiHelsinki, Finland

**Keywords:** *E. faecium*, *E. faecalis*, high-risk nosocomial clones, antibiotic resistance, virulence

## Abstract

Enterococci are Gram-positive bacteria that are found in plants, soil and as commensals of the gastrointestinal tract of humans, mammals, and insects. Despite their commensal nature, they have also become globally important nosocomial pathogens. Within the genus *Enterococcus, Enterococcus faecium*, and *Enterococcus faecalis* are clinically most relevant. In this review, we will discuss how *E. faecium* and *E. faecalis* have evolved to become a globally disseminated nosocomial pathogen. *E. faecium* has a defined sub-population that is associated with hospitalized patients and is rarely encountered in community settings. These hospital-associated clones are characterized by the acquisition of adaptive genetic elements, including genes involved in metabolism, biofilm formation, and antibiotic resistance. In contrast to *E. faecium*, clones of *E. faecalis* isolated from hospitalized patients, including strains causing clinical infections, are not exclusively found in hospitals but are also present in healthy individuals and animals. This observation suggests that the division between commensals and hospital-adapted lineages is less clear for *E. faecalis* than for *E. faecium*. In addition, genes that are reported to be associated with virulence of *E. faecalis* are often not unique to clinical isolates, but are also found in strains that originate from commensal niches. As a reflection of more ancient association of *E. faecalis* with different hosts, these determinants Thus, they may not represent genuine virulence genes but may act as host-adaptive functions that are useful in a variety of intestinal environments. The scope of the review is to summarize recent trends in the emergence of antibiotic resistance and explore recent developments in the molecular epidemiology, population structure and mechanisms of adaptation of *E. faecium* and *E. faecalis*.

## Introduction

Enterococci are low-GC Gram-positive ovococci that can form pairs and chains of diverse lengths. Bacteria from the genus *Enterococcus* are facultative anaerobic and grow optimally at 35°C, but can tolerate temperatures ranging from 10°C to 45°C (Byappanahalli et al., 2012). The genus comprises 54 species (Parte, 2014), which are ubiquitously present in nature but the gastrointestinal tract (GIT) of animals, including mammals, reptiles, birds (Mundt, 1963a) and insects (Martin and Mundt, 1972), is thought to be the largest reservoir of enterococci (Gilmore et al., 2013).

In humans, enterococci are common commensals of the GIT. In addition, enterococci have become ever more prominent as a causative agent of nosocomial infections since the 1970s (Arias and Murray, 2012). Two species, *Enterococcus faecalis* and *Enterococcus faecium*, cause the vast majority of hospital-acquired enterococcal infections in humans (Agudelo Higuita and Huycke, 2014). Of these two, *E. faecium* has rapidly acquired resistance to several classes of antibiotics. First, in the 1970s and 1980s, *E. faecium* gained high-level resistance to ampicillin (Grayson et al., 1991; Galloway-Peña et al., 2009) and since the 1980s it acquired resistance to aminoglycosides, fluoroquinolones, and glycopeptides, particularly vancomycin (Leclercq et al., 1988; Uttley et al., 1988; National Nosocomial Infections Surveillance System, 2004). *E. faecalis* has also acquired resistance to aminoglycosides, but resistance to ampicillin and vancomycin is much rarer than in *E. faecium* (Edelsberg et al., 2014). Worryingly, resistance to antibiotics that are used to treat vancomycin resistant enterococci (VRE), like linezolid, tigecycline, and daptomycin, has already been reported (Aksoy and Unal, 2008; Montero et al., 2008; Scheetz et al., 2008; Niebel et al., 2015).

The intrinsic resistance of enterococci to some antibiotics, including aminoglycosides, and the ability to acquire and disseminate antibiotic resistance determinants, like those involved in vancomycin resistance, only partly explain the recent emergence of these organisms as nosocomial pathogens. In addition, the plasticity of the enterococcal genomes allow enterococci to rapidly respond and adapt to selective constraints by acquiring genetic determinants that increase their ability to colonize or infect the host (Hendrickx et al., 2009; Palmer et al., 2012; Lebreton et al., 2013; Van Tyne and Gilmore, 2014). Other host or environmental factors, most notably exposure to antimicrobial agents, may favor an increase in colonization density of enterococci in the GIT of hospitalized patients (Donskey et al., 2000; Ubeda et al., 2010; Ruiz-Garbajosa et al., 2012). Antibiotic therapy that leads to the depletion of Gram-negatives, can reduce production of the antimicrobial peptide REGIIIγ by Paneth cells, and this may promote the outgrowth of VRE (Brandl et al., 2008).

Patients undergoing transplants or with underlying diseases, such as diabetes or renal failure, and patients with long-term catheter usage, are at higher risk of developing infections caused by multi-drug resistant (MDR) enterococci (Arias and Murray, 2012). High-density colonization of the patient GIT facilitates the transmission of MDR enterococci among hospital ward through fecal contamination (Arias and Murray, 2012). Therefore epidemiological surveillance and outbreak investigations, together with infection control polices and interventions, such as the use of protective barriers and proper disinfection, are key for infection control of these organisms in the nosocomial environment (Sydnor and Perl, 2011). High-level enterococcal GIT colonization can also lead to urinary tract infections (UTI) (De Vecchi et al., 2013; Neelakanta et al., 2015), which may progress to bloodstream infections or endocarditis (Patterson et al., 1995; Fernández-Guerrero et al., 2002). Enterococci from high-density intestinal populations may also directly translocate from the GIT into the bloodstream (Kamboj et al., 2014).

Enterococci have become one of the most common causes of health care-associated infections with *E. faecalis* causing approximately 60% of infections and *E. faecium* the remainder (de Kraker et al., 2013; Sievert et al., 2013). This review will focus on *E. faecalis* and *E. faecium* as both species have emerged as important nosocomial pathogens over the last 30 years and represent a major hub for the dissemination of antibiotic resistance genes.

## Emergence of Antibiotic Resistance in Enterococci

*Enterococcus faecalis* and *E. faecium* exhibit intrinsic resistance to a broad range of antibiotics. Below we briefly describe the mechanisms that cause resistance to the classes of antibiotics that are currently used in the treatment of enterococcal infections.

Compared to other low-GC Gram-positive cocci, enterococci exhibit decreased susceptibility to β-lactam antibiotics. The β-lactams act through the inactivation of penicillin-binding proteins (PBPs), thereby interfering with synthesis of peptidoglycan. All enterococci display decreased susceptibility to β-lactam antibiotics due to the expression of PBPs with an intrinsic low affinity for this class of antibiotics. Resistance to β-lactams, most notably to ampicillin, is currently far more widespread in *E. faecium* than in *E. faecalis* (Cattoir and Giard, 2014). The most important determinants for β-lactam resistance in *E. faecium* are mutations in the genes encoding the PBP5 (Zorzi et al., 1996; Rice et al., 2001; Zhang et al., 2012). There are clear indications that *E. faecium* progressed toward high-level ampicillin resistance in the 1970s and 1980s through the acquisition of specific mutations in the *pbp5* gene (Grayson et al., 1991; Galloway-Peña et al., 2009, 2011). It is of importance to note that these chromosomally encoded PBPs can be transferable (Dahl et al., 2000; Hanrahan et al., 2000; Rice et al., 2005), which indicates that dissemination of high-level ampicillin resistance can be the result of both clonal spread of strains with mutated *pbp5* genes and horizontal gene transfer. In addition to mutations in *pbp5*, production of β-lactamase has been described in both *E. faecalis* and *E. faecium* (Tomayko et al., 1996; Sarti et al., 2012). In general, the expression level of beta-lactamase in these species is low and impact on ampicillin susceptibility marginal.

Another group of antibiotics to which *E. faecium* and *E. faecalis* exhibit moderate intrinsic and high-level acquired resistance are the aminoglycosides. In *E. faecalis*, intrinsic resistance is thought to be caused by the inability of the antibiotic to enter the cytoplasm and inhibit ribosomal protein synthesis (Aslangul et al., 2006). In *E. faecium*, two chromosomally encoded genes, a 6′-*N*-aminoglycoside acetyltransferase (*aac(6*′*)-Ii*) (Costa et al., 1993) and an rRNA methyltransferase (*efmM*) (Galimand et al., 2011), have been associated with intrinsic resistance to tobramycin and kanamycin. In addition to intrinsic resistance to aminoglycosides, the therapeutic success of these antibiotics is critically compromised by high-level resistance, due to the gain of aminoglycoside modifying enzymes, such as phosphotransferases, acetyltransferases, and nucleotidyltransferases by *E. faecium* and *E. faecalis* (Chow, 2000; Miller et al., 2014).

The assessment of enterococci, particularly *E. faecium*, as important agents of MDR nosocomial infections was definitively established when they acquired resistance to vancomycin. While vancomycin-resistant enterococci were virtually non-existent in hospitals in the USA before 1990, nowadays 87% of *E. faecium* strains from nosocomial infections are vancomycin-resistant, while this is only 14% for *E. faecalis* (Edelsberg et al., 2014). Vancomycin is a glycopeptide antibiotic, which prevents cross-linking of peptidoglycan by binding to the D-alanine-D-alanine (D-Ala-D-Ala) moiety of the peptide chains that crosslink peptidoglycans. The mechanisms by which enterococci become resistant to vancomycin have been extensively reviewed elsewhere (Courvalin, 2006). In short, enterococci become resistant to vancomycin when the terminal amino acids of peptidoglycan precursors are altered from D-Ala-D-Ala to D-Ala-D-lactate (D-Ala-D-Lac) or to D-Ala-D-Serine (D-Ala-D-Ser), leading to high-level and low-level resistance to vancomycin, respectively. Nine gene clusters are currently known to be involved in vancomycin resistance in enterococci. These vancomycin resistance gene clusters are *vanA, vanB, vanD*, and *vanM*, causing vancomycin resistance through the formation of D-Ala-D-Lac, and *vanC, vanE, vanG, vanL*, and *vanN* which catalyze the formation of D-Ala-D-Ser (Courvalin, 2006; Boyd et al., 2008; Xu et al., 2010; Lebreton et al., 2011). The most frequently found, and thus clinically most relevant, vancomycin resistance determinants are *vanA* and *vanB*, which are both part of larger gene clusters, which encode a two-component regulatory system and enzymes involved in the recycling of D-Ala-D-Ala peptidoglycan precursors to D-Ala-D-Lac. Both *vanA* and *vanB* are located on transposons that contribute to the dissemination of vancomycin resistance among enterococci (Courvalin, 2006). Recently, *vanM* was found to be the most important vancomycin-resistance determinant among different *E. faecium* lineages in hospitals in Shanghai, China (Chen et al., 2015). Differences in the sequence diversity and prevalence of each *van* operon could be due to the different ecological origins of the *van* clusters. While *vanA* seems to have originated from soil organisms, *vanB, vanG*, and *vanD* have been reported in gut commensal microbiota (Guardabassi et al., 2005; Domingo et al., 2007; Howden et al., 2013).

While vancomycin resistance emerged and spread in USA hospitals in the 1990s, carriage of vancomycin-resistant *E. faecium* (VREF) was rare among hospitalized patients in Europe. In contrast, VRE carriage among farm animals, and, to a lesser extent, in healthy humans was higher in Europe than in the USA (Klare et al., 1995; Devriese et al., 1996; Stobberingh et al., 1999). The widespread occurrence of VRE among farm animals was linked to the use of the vancomycin-analog avoparcin as a growth promoter in Europe (Bager et al., 1997). The presence of indistinguishable vancomycin resistance transposons in both animal and human reservoirs provided the first indication that animal-derived enterococci could be a reservoir of antibiotic resistance genes that could be transmitted to humans (Arthur et al., 1993; Jensen et al., 1998; Woodford et al., 1998; Willems et al., 1999). After the ban on the use of avoparcin in 1997, the prevalence of VRE in animal husbandry declined in Europe (Klare et al., 1999; van den Bogaard et al., 2000; Aarestrup et al., 2001). In several European countries, the prevalence of VRE among hospitalized patients increased in the 21st century, but is still lower than the endemic levels reported in US hospitals (Bourdon et al., 2011; Gastmeier et al., 2014; Pinholt et al., 2015; Simner et al., 2015). Levels of VRE among hospitalized patients are also high in Australia, with 37% of *E. faecium* bacteremia isolates exhibiting resistance to vancomycin. Interestingly, vancomycin resistance in Australian *E. faecium* isolates is almost exclusively caused by *vanB*-type transposons, while *vanA* is the major vancomycin-resistance determinant in Europe and the USA (Coombs et al., 2014).

Due to the emergence and rapid spread of VRE, the antibiotics linezolid, daptomycin and tigecycline are increasingly used for the treatment of VRE infections.

Linezolid, the first oxazolidinone antibiotic, was introduced for clinical use in the USA in 2000. It acts on the ribosome by binding to a universally conserved site on 23S rRNA of the large 50S subunit of the ribosome (Colca et al., 2003), thereby inhibiting elongation of the polypeptide chain. The currently described linezolid resistance mechanisms alter the peptidyl transferase center of 23S rRNA, by a mutation in the 23S rRNA gene, with the G2576T mutation being most prominent (Jones et al., 2008), mutations in the genes encoding ribosomal proteins L3 and L4 (Chen et al., 2013) or methylation of the adenine at position 2503, which is catalyzed by the methyltransferase Cfr. The *cfr* gene is encoded on various conjugative and non-conjugative plasmids in enterococci (Diaz et al., 2012; Shen et al., 2013). Recently, the oxazolidinone resistance gene *optrA* has been identified in *E. faecalis* and *E. faecium* isolates of human and animal origin (Cai et al., 2015; Wang et al., 2015). This gene codes for an ATP-binding cassette transporter, which contributes to reduced susceptibility for oxazolidinones (linezolid and tedizolid) and phenicols (chloramphenicol and florfenicol). The *optrA* gene was associated with a conjugative plasmid (Wang et al., 2015). Nevertheless, linezolid resistance is still rare. In a study using data from 19 US hospitals in the period 2007–2010, linezolid resistance was reported in 1.1 and 1.8% of *E. faecium* and *E. faecalis* isolates, respectively (Edelsberg et al., 2014).

Daptomycin is a lipopeptide antibiotic that targets the cell membrane through interactions with phospholipids (Humphries et al., 2013). The genetic basis for resistance in *E. faecalis* and *E. faecium* was first studied through genome sequencing of pairs of strains that developed resistance *in vitro* or *in vivo* (Arias et al., 2011; Palmer et al., 2011; Tran et al., 2013a). Additional genetic and biochemical work has established the role of the three-component regulatory system LiaFSR in contributing to daptomycin resistance in enterococci. The LiaFSR system is conserved in low-GC Gram-positive bacteria and governs the cell envelope stress response (Jordan et al., 2006). Mutations in the *liaF* gene of *E. faecalis* lead to a redistribution of cardiolipin-rich microdomains from the division septum to other regions of the cytoplasmic membrane, which affects the antimicrobial activity of daptomycin. In addition, other mutations in phospholipid biosynthesis genes, most notably in the cardiolipin synthase gene *cls*, are required for full expression of daptomycin resistance in *E. faecalis* (Davlieva et al., 2013; Tran et al., 2013b). Similar mechanisms have been associated with daptomycin resistance in *E. faecium* (Munita et al., 2012, 2014; Davlieva et al., 2013). Notably, emergence of daptomycin resistance in *E. faecium* during daptomycin therapy is not always linked to mutations in *liaFSR* and mutations in *cls* may already be sufficient (Kelesidis et al., 2013; Lellek et al., 2015). Daptomycin resistance is infrequent, but more common in *E. faecium* than in *E. faecalis.* A worldwide study in isolates from hospitalized patients over the period 2005–2012 showed levels of daptomycin resistance of 0.18% for *E. faecium* and 0.02% for *E. faecalis* (Sader et al., 2014). However, in certain settings daptomycin resistance may be considerably more frequent, as illustrated by a study including 4,274 *E. faecium* and 7,007 *E. faecalis* isolates from 19 US hospitals, which found daptomycin resistance among 3.9 and 0.2% of *E. faecium* and *E. faecalis* isolates, respectively (Edelsberg et al., 2014). Similar levels of daptomycin resistance in *E. faecium* were recently reported for a German hospital (Lübbert et al., 2015).

Tigecycline is a semisynthetic derivative of the broad-spectrum tetracycline antibiotic minocycline, which acts on the ribosome by inhibiting its association with aminoacyl-tRNAs. The emergence of resistance during tigecycline therapy was first observed in *E. faecalis* (Werner et al., 2008; Cordina et al., 2012) and has recently been described in *E. faecium* (Niebel et al., 2015). In enterococci, resistance to tigecycline can be mediated through upregulation of tetracycline resistance determinants *tetL* (encoding an efflux pump) and *tetM* (providing ribosomal protection) (Fiedler et al., 2015) and mutations in the ribosomal protein *rpsJ* (Beabout et al., 2015; Cattoir et al., 2015; Niebel et al., 2015). Tigecycline resistance among *E. faecium* and *E. faecalis* is rare at 0.3% for both species (Hoban et al., 2015) and the antibiotic can still be used successfully to treat bacteremias caused by MDR enterococci, especially when it is used in combination with daptomycin (Polidori et al., 2011). The recovery of enterococcal isolates with decreased susceptibility to tigecycline in samples of animal origin is of concern, as extensive use with tetracyclines in the veterinary setting could select for tigecycline tolerant strains (Freitas et al., 2011).

In addition to the antibiotics described above, the antibiotics tedizolid, telavancin, dalbavancin, and oritavancin have recently been approved by the Food and Drug Administration in the USA for the treatment of skin infections by Gram-positive bacteria (Crotty et al., 2016). Tedizolid is an oxazolidinone antibiotic that has improved *in vitro* activity compared to linezolid. Enterococcal strains that have acquired linezolid resistance due to the acquisition of the *cfr* gene or the G2576T mutation in the 23S rRNA gene, may still have relatively low MICs (≤4 μg/ml) for tedizolid (Barber et al., 2016; Silva-Del Toro et al., 2016). Telavancin, dalbavancin and oritavancin are semi-synthetic glycopeptides antibiotics that, similar to vancomycin, affect peptidoglycan stability by binding to the D-Ala-D-Ala moiety of the peptide chains that crosslink peptidoglycan chains. Interestingly, oritavancin is also active against *vanA-* and *vanB-*type vancomycin-resistant enterococci, while telavancin and dalbavancin have limited activity against enterococci carrying the *vanA*-type vancomycin resistance transposon but are active against enterococci with *vanB-*type vancomycin resistance (Jones et al., 2013; Karlowsky et al., 2015). Tedizolid, telavancin, dalbavancin, and oritavancin may be useful as alternatives to linezolid and vancomycin, but they do not have radically different modes of action and they may therefore suffer from the same resistance mechanisms that are threatening the efficacy of linezolid and vancomycin. Therefore, antibiotics that target other structures in the enterococcal cell, including teixobactin (Ling et al., 2015) and the acyldepsipeptides (Brötz-Oesterhelt et al., 2005), hold considerable promise as novel compounds for the treatment of infections with MDR enterococci.

## Molecular Epidemiology and Population Structure of *E. faecium* and *E. faecalis*

### E. faecium

As a consequence of the global rise of VREF, a large number of molecular epidemiological studies have been performed to obtain insights into the dissemination of VREF clones in and between hospitals, in farm animals and healthy humans. However, methods like pulse-field gel electrophoresis (PFGE), soon proved to be insufficiently reproducible to study genetic relatedness of isolates (Morrison et al., 1999).

A first insight into the existence of particular ecotypes in *E. faecium*, was obtained by using amplified fragment polymorphism (AFLP) to infer the genetic relatedness of strains from diverse hosts and environments (Willems et al., 2000). This study revealed that strains from hospitalized patients grouped in a specific sub-population that was distinct from groups of strains that were isolated from humans in the community and farm animals.

The use of AFLP for global studies showed the limitations of this technique for comparisons of data obtained from different laboratories. Therefore an alternative method, termed multi locus sequence typing (MSLT), was used in follow-up studies. In MLST, allelic profiles are based on the sequences of fragments of a number of housekeeping genes (seven in the case of the *E. faecium* and *E. faecalis* MLST schemes) (Homan et al., 2002; Ruiz-Garbajosa et al., 2006). Compared to AFLP, MLST has the distinct advantage that data are easily collated and shared through an on-line database. The first analyses of *E. faecium* MLST data were performed with the algorithm eBurst (Feil et al., 2004) and confirmed a distinct clustering of strains derived from the hospital environment. This cluster was named clonal complex 17 (CC17) and soon CC17 was found to be disseminated throughout the world (Willems et al., 2005; Top et al., 2008). The later use of larger datasets and different algorithms for the analysis of MLST data (Didelot and Falush, 2007; Francisco et al., 2009), showed that eBurst can sometimes fail to correctly assign STs to clonal complexes (CCs), particularly when genetic variation in populations is largely driven by recombination rather than mutations, like is the case in *E. faecium* and *E. faecalis* (Turner et al., 2007).

Bayesian analysis of population structure (BAPS) has successfully been used to probabilistically infer the population structure and levels of recombination of several microbial pathogens (Corander et al., 2012; Thomas et al., 2014). When applied to *E. faecium* MLST data, BAPS allowed the partitioning of 519 STs of 1720 *E. faecium* isolates into 13 non-overlapping groups. Of these groups, BAPS 3–3 was significantly associated with isolates from hospitalized patients, while BAPS 2–1 and 2–4 were significantly associated with farm animals. This observation again confirmed that there exists structure in the *E. faecium* population, with a distinct subpopulation of isolates that are almost exclusively found in hospitalized patients (Willems et al., 2012). One of the important nodes in the previously described hospital-associated CC17, ST78, and its descendant STs, grouped in BAPS 2–1 together with farm animal isolates, while two other important CC17 nodes, ST17 and ST18 with their descendant STs, clustered in another BAPS group (BAPS 3–3). These findings indicate that nosocomial *E. faecium* isolates have not evolved from a single ancestor, like previously postulated, but rather the cumulative acquisition of adaptive elements in nosocomial isolates may have occurred multiple times in different genetic backgrounds. Another conclusion that could be drawn from Bayesian modeling of MLST data is that hospital isolates displayed a relative low-level of admixture, despite the high recombination rates in *E. faecium*, suggesting that once strains have adapted to the distinct hospital niche, they become ecologically isolated (including isolation by dominance) and recombination with other populations declines (Willems et al., 2012).

Despite the overt lack of reproducibility, PFGE long remained the “gold standard” for molecular typing of *E. faecium* until the introduction of whole genome sequence (WGS)-based epidemiology. Howden et al. (2013) published a landmark study in which they used whole genome sequencing to track an outbreak of *vanB*-VREF in a large hospital in Australia. Interestingly, detailed phylogenomic analysis and precise mapping of the *vanB* gene revealed that 18 of the 36 *vanB*-VREF had acquired the *vanB* transposon during the outbreak period. This indicates that for *vanB*-VREF, frequent *de novo* generation of VREF through horizontal gene transfer may contribute to the emergence of VREF, in addition to clonal spread. This study was followed by multiple other studies that used whole genome sequencing to investigate the molecular epidemiology of VREF with high resolution and high accuracy (Reuter et al., 2013; Salipante et al., 2015; Brodrick et al., 2016; van Hal et al., 2016). Bender et al. (2016) performed whole genome sequencing of 49 *vanB*-VREF, primarily of ST192 (39/49), from invasive infections from hospitals all across Germany and found that spread of the Tn*1549-vanB*-type resistance involved exchange of large chromosomal fragments between *vanB*-positive and *vanB*-negative enterococci rather than independent acquisition events of the *vanB* transposon alone.

**FIGURE 1:**
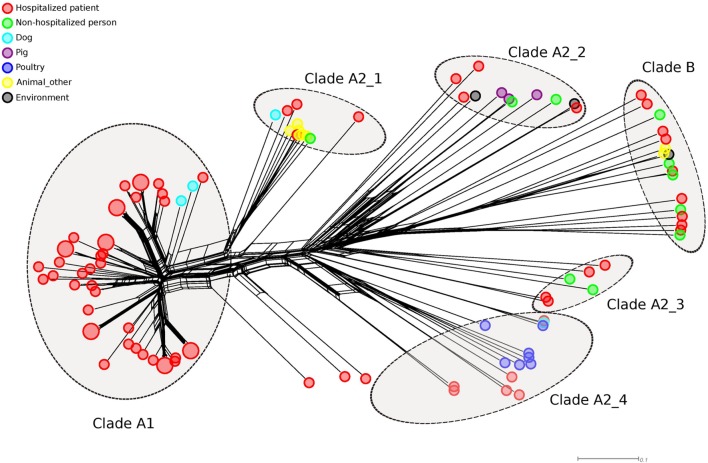
**NeighborNet phylogenetic network to visualize the relationships between 196 *E. faecium* isolates.** The distance matrix underlying the network was built from all pairwise allelic profile comparisons of 1423 loci of the published *E. faecium* cgMLST scheme (de Been et al., 2015). Allelic profiles were extracted from SeqSphere. Small colored circles indicate the origin of isolates. *E. faecium* clades (A1 and B) inferred from the STs and based on Lebreton et al. (2013) are indicated by large dotted circles. Notably, in this analysis clade A2 isolates do not group in a single clade but form at least four distinct clusters that are located in the network and are indicated clade A2_1, A2_2, A2_3 and A2_4.

WGS-based studies also proposed that the *E. faecium* population was divided into two species-level subdivisions, based on phylogenetic analysis and the determination of average nucleotide identity (ANI) between the two sub-populations. The sub-populations were termed clade A or hospital-associated clade, primarily containing isolates from hospitalized patients and clade B or community-associated clade, mostly containing isolates from healthy, non-hospitalized individuals (Galloway-Peña et al., 2012; Palmer et al., 2012). The high level of diversity between these two clades indicates that the clade A–B split is ancient and precedes the modern antibiotic era. Further work provided further evidence for this split and showed that the population of *E. faecium* in clade A had a second split of more recent date (74 ± 30 years), with clade A1 containing the majority of clinical isolates and clade A2 mostly comprising animal-derived strains (Lebreton et al., 2013). WGS also confirmed that *E. faecium* was subject to high rates of recombination, leading to changes in MLST profile in otherwise closely related strains, which invalidates the use of MLST for tracking transmission events (van Hal et al., 2016). Another finding of van Hal et al. (2016) was that other *E. faecium* strains are the most important donors of imported DNA fragments. Specifically, strains from clade B are an important reservoir for donating foreign DNA to clade A strains (de Been et al., 2013). These findings indicate that hospital-acquired clade A1 *E. faecium* strains have recently emerged from a background of human commensal and animal isolates. Tree-like network representations of *E. faecium* genomic relatedness based on cgMLST data also revealed the distinct clustering of human commensal, animal and human clinical strains (**Figure 1**). Notably, in this analysis the animal isolates do not group in a single clade A2 but form multiple distinct clusters that are located in the network, between the human commensal and clinical isolates, indicating that clade A2 may not be monophyletic, as previously postulated (Lebreton et al., 2013). Interestingly, clade A1 *E. faecium* strains have lower fitness in natural environments, where they are out-competed by other *E. faecium* clones (Leclercq et al., 2013). Similarly, clade B strains outcompete clade A strains in an animal model of gut colonization in the absence of selection by antibiotics (Montealegre et al., 2015). These data highlight the exquisite niche specialization to the hospital environment of clade A1 strains, which may come at a fitness cost in non-hospital environments.

The WGS-based epidemiological studies described above generated phylogenetic trees based on SNPs, by mapping sequencing reads to a reference genome. This approach, although providing high-resolution typing data, complicates comparisons of data between studies and hampers the construction of globally accessible databases. This limitation may be overcome by using a genome-wide gene-by-gene comparison approach, as in classical MLST, but with an important extension of the number of analyzed genes from seven to the entire core genome of the species, i.e., 1423 genes as in the recently published core genome MLST (cgMLST) scheme of *E. faecium* (de Been et al., 2015). The *E. faecium* cgMLST scheme allowed high-resolution tracing of *E. faecium* outbreaks and performed equally well as an SNP-based phylogenetic approach, but has the advantage that data can be collated in an online database and a standardized nomenclature for clones can be used.

### E. faecalis

*Enterococcus faecalis* appears to be the most widespread and abundant species of *Enterococcus* and can be found in the intestines of humans, farm, companion, and wild animals and in the environment (Mundt, 1963a,b; Devriese et al., 1992a,b; Tannock and Cook, 2002; Ruiz-Garbajosa et al., 2009). Initial molecular epidemiological studies, using MLST, showed the existence of CCs, in which isolates originating from different hosts are contained (Ruiz-Garbajosa et al., 2006; McBride et al., 2007). While many *E. faecalis* clones are shared between hospitalized patients and other reservoirs, some CCs (specifically CC2, CC9, and CC87) and STs (ST6 and ST16) seemed to be enriched among isolates from hospitalized patients (Ruiz-Garbajosa et al., 2006; McBride et al., 2007; Freitas et al., 2009; Kuch et al., 2012). Similarly, in isolates collected from across the globe, antibiotic resistance is more prevalent in strains that belong to CC2, CC8, CC9, CC16, and CC87 (Kawalec et al., 2007; McBride et al., 2007; Freitas et al., 2009; Kuch et al., 2012).

Analysis of the extent of congruence between the topologies of the seven different MLST gene trees revealed that that all 42 pairwise comparisons of the sequences of the MLST loci were incongruent. This observation suggests that *E. faecalis* is a highly recombinogenic species (Ruiz-Garbajosa et al., 2006). For this reason Tedim et al. (2015) used BAPS to investigate the *E. faecalis* population structure based on MLST data of hospitalized (*n* = 133) and non-hospitalized individuals (*n* = 173) isolated from feces. A hierarchical BAPS clustering analysis partitioned the *E. faecalis* population into 5 BAPS groups. Contrary to what has been described in *E. faecium* and to eBURST based analyses of *E. faecalis* MLST data, no significant association was found between isolates from hospitalized patients and particular *E. faecalis* BAPS groups (Tedim et al., 2015).

**FIGURE 2:**
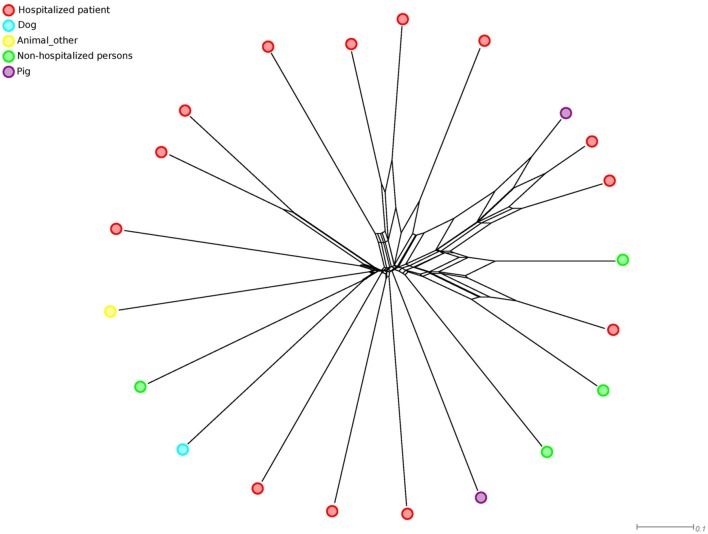
**NeighborNet phylogenetic network to visualize the relationships between 21 *E. faecalis* isolates.** The distance matrix underlying the network was built from all pairwise allelic profile comparisons of 1298 loci of an *ad hoc E. faecalis* cgMLST scheme using SeqSphere (http://www.ridom.de/seqsphere/). Small colored circles indicate the origin of isolates.

The first comprehensive *E. faecalis* phylogenomic study involved 18 *E. faecalis* genomes representing clinical (*n* = 10), human commensal (*n* = 3), and animal isolates (*n* = 3), and isolates of unknown origin (*n* = 2) (Palmer et al., 2012). The analysis revealed that the phylogenetic diversity of *E. faecalis* was limited compared to *E. faecium*, with variations in the ANI in a narrow range between 97.8 and 99.5%. There was also no clear structure in the phylogenetic core gene tree, with no distinct clustering of isolates according to source. Similarly, clustering of these 18 isolates supplemented with 3 other published *E. faecalis* genomes from a clinical site, human feces and pig based on an *ad hoc* developed *E. faecalis* cgMLST scheme using tree-like network methods also revealed no distinct clustering of isolates according to their source (**Figure 2**). A recent genome-based study of 515 *E. faecalis* genomes, mainly isolated from clinical settings on the British Isles, revealed three dominant lineages of hospital-associated lineages (L1, L2, and L3). Isolates in L1 and L3 originated from both the UK and USA (Raven et al., 2016). These data clearly indicate independent clonal expansion with subsequent national dissemination. The data may also point toward the existence of specific hospital-associated or hospital-adapted lineages. However, in order to unequivocally infer hospital-adapted lineages it is essential to correct the data for clonal outbreaks, by including outbreak clones only once in the analysis. The fact that only a limited number of STs (3, 2, and 2 STs in lineage 1, 2, and 3, respectively) were represented in these three lineages may suggest that these lineages contain multiple replicates of outbreaks clones. Analyses for recombination frequencies in the core genome of L1, L2 and L3 isolates indicated low levels of recombination, which seems to contrast with previous MLST-based analyses (Ruiz-Garbajosa et al., 2006; Raven et al., 2016). Possibly, recombination drove the initial diversification of *E. faecalis* but contributed less to the relatively recent evolution of the three dominant *E. faecalis* lineages described by Raven and co-workers (Raven et al., 2016).

## Adaptive Elements in Nosocomial Lineages

Enterococci are ubiquitous in nature, where they act as commensals and opportunistic pathogens. A consequence of these different lifestyles is that enterococci need to adapt to different micro-environments, each one exerting strong selective pressures. A strategy for bacterial species to survive when confronted with a wide range of selective pressures is to specialize in particular fitness peaks. *E. faecium* probably followed such an evolutionary trajectory, resulting in the emergence of specific hospital-adapted lineages. Successful hospital-adapted clones in *E. faecium* have the ability to exchange mobile genetic elements, carrying antimicrobial resistance, and virulence determinants, by horizontal gene transfer (Leavis et al., 2003; Hegstad et al., 2010; Palmer et al., 2010; van Schaik et al., 2010). The cumulative acquisition of adaptive elements has been named “genetic capitalism”, in which the acquisition of a particular adaptive element by a particular clone enhances its fitness, thereby increasing the likelihood of acquiring a second adaptive element which finally can lead to the emergence of high-risk multidrug-resistant clones (Baquero, 2004). This cumulative acquisition of adaptive elements by the hospital-associated *E. faecium* clade A1 strains is reflected by their larger genome sizes (2,843 ± 159 genes; 2.98 ± 0.15 Mbp), compared to the genomes of strains in clade A2 (2,597 ± 153 genes; 2.75 ± 0.14 Mbp) or clade B (2,718 ± 120 genes; 2.84 ± 0.1 Mbp) (Lebreton et al., 2013).

The first adaptive element that was described as being specific for hospital-associated *E. faecium* strains was Esp (Willems et al., 2001), a surface protein with a signal sequence for transport and a LPxTG-like motif for cell wall anchoring. The *E. faecium esp* gene is located on an integrative conjugative element, called ICE*Efm1*, and contributes to biofilm formation, and UTIs and endocarditis in animal models (Leavis et al., 2004; Heikens et al., 2007, 2011; Leendertse et al., 2009; Sava et al., 2010; van Schaik et al., 2010; Top et al., 2011).

In addition to Esp, other determinants were found to be specific or significantly enriched among hospital-associated isolates. Characteristics of these genes have been previously discussed (Leavis et al., 2003; Heikens et al., 2008; Hendrickx et al., 2009, 2013; Zhang et al., 2013; Paganelli et al., 2016). Comparative genomic analyses of 73 *E. faecium* strains revealed major differences in gene content between clinical (clade A1), animal (clade A2) and non-clinical (clade B) strains, particularly through gain and loss of gene clusters with predicted roles in carbohydrate metabolism (Kim and Marco, 2013; Lebreton et al., 2013). Many of the clade B-specific genes have a predicted role in the utilization of complex carbohydrates from dietary sources, which were replaced with genes that were associated with the utilization of amino sugars (e.g., galactosamine), which occur on epithelial cell surfaces and in mucin. This metabolic switch may have contributed to the niche specialization of *E. faecium*.

As mentioned above the population structure of *E. faecalis* seems to be significantly different from *E. faecium* as no clearly defined *E. faecalis* ecotypes appear to exist. Correspondingly, previous studies have reported shared antibiotic resistance and virulence genes, such as the *E. faecalis* pathogenicity island, *esp*, capsule polysaccharide genes, and genes encoding for gelatinase, aggregation substance, cytolysin, and Ace, among *E. faecalis* isolates from a wide variety of different niches like the hospital, animals, food products and the environment (Eaton and Gasson, 2001; Creti et al., 2004; McBride et al., 2007; Freitas et al., 2009; Solheim et al., 2009; La Rosa et al., 2015). Note that the absence of clearly defined ecotypes in *E. faecalis* can be interpreted as a high multiplicity of closely related fine-grained ecotypes, none of them reaching predominance.

Comparative genomic analysis of 18 *E. faecalis* genomes showed that the accretion of mobile genetic elements in multiple *E. faecalis* lineages appears to be a major source of genome diversity (Palmer et al., 2012). Although antibiotic resistance and pathogenicity island traits have converged in some *E*. *faecalis* lineages, substantial differences in gene content exist, indicating that specialization toward specific ecotypes is not apparent in this species, in contrast to *E. faecium*. In addition, a hierarchical clustering based on genome content of 38 *E. faecalis* genomes from diverse sources including clinical and non-clinical isolates, unearthed no evidence of distinct lineages in *E. faecalis* and no genes were found to be significantly enriched among clinical or non-clinical strains (Kim and Marco, 2013). A pangenome analysis of 168 *E. faecalis* isolates revealed no genes, or even homoplastic non-synonymous single nucleotide polymorphisms, that were ubiquitous in the three dominant lineages of hospital isolates, while being absent from all other sporadic lineages (Raven et al., 2016). These observations are all in accordance with high levels of genetic exchange between ecologically diverse *E. faecalis* clones.

In bacteria, mechanisms that preclude the acquisition of foreign DNA, include the Clustered Regularly Interspaced Short Palindromic Repeats (CRISPR)-Cas system (Horvath and Barrangou, 2010) and restriction and anti-restriction modification (RM-, antiRM-) systems (Clewell et al., 2014). *E. faecalis* strains lacking CRISPR-Cas appear to more readily acquire DNA through horizontal gene transfer and, consequently have a larger genome size than *E. faecalis* strains that carry CRISPR-Cas (Palmer et al., 2010, 2012). This indicates that the CRISPR-Cas status of *E. faecalis* strains may contribute to ecological adaptation. In *E. faecium* CRISPR-Cas is only found in a subset of clade B strains, again highlighting the general lack of barriers to horizontal gene exchange in this species (Lebreton et al., 2013). Restriction and anti-restriction systems located on mobile elements or enterococcal chromosomes can also determine the flow of adaptive traits among populations of the same and/or different species (Clewell et al., 2014). Anti-RM systems include analogs of ArdA (alleviation of restriction of DNA) that are located on conjugative transposons (CTns) that are found widely spread in enterococci. ArdA proteins act against Type I restriction systems (detected in Tn*916* and CTn*6000*) and other genes presumptively involved in methylation (CTn*6000*) (Clewell et al., 2014). Recently, a type IIS restriction-modification (R-M) system SfaNI was described in *E. faecalis* (Furmanek-Blaszk and Sektas, 2015).

Other strategies seem to have exerted positive as well as negative selective pressures on *E. faecalis* clones. *E. faecalis* strain V583 uses phage particles to establish and maintain dominance of its intestinal niche in the presence of closely related competing strains (Duerkop et al., 2012). Furthermore, five of the seven prophages in the same strain (phage01, phage03, phage04, phage05, and phage07) can be excised from the bacterial chromosome and four of them produced infective virions that may promote gene dissemination among isolates and increase pathogenicity (Matos et al., 2013; La Rosa et al., 2015). Conversely, Gaca and Gilmore recently demonstrated how the accretion of mobile genetic elements in *E. faecalis* V583, renders it unable to co-exist with native enterococci in healthy human fecal flora (Gaca and Gilmore, 2016).

## Conclusion

The ubiquitous nature of enterococci, the flexibility of their genomes and the widespread use of antibiotics in human and veterinary medicine, are important factors that drive the current emergence of *E. faecium* and *E. faecalis* as MDR nosocomial pathogens.

The population structure of *E. faecium* is characterized by a deep phylogenetic split that separates human commensal isolates (clade B) from farm animal and hospital isolates (clade A). It is tempting to speculate that the deep phylogenetic split in *E. faecium* is driven by anthropogenic influences since strains from farm animals (clade A2) and clinical isolates (clade A1), share a common feature as they both originate from environments where mammalian hosts are in close contact with each other and usage of antibiotics is high. The evolutionary trajectory of *E. faecium*, which has led to a clear clade structure, suggests that *E. faecium* is colonizing rugged fitness landscapes, in which characters of particular clones in clade A and B impose strong fitness differences. Under these circumstances, gene exchange between diverging populations is reduced over large genomic regions, as a collateral effect of strong divergent selection on genes involved in local adaptation. This mechanism was named divergence hitchhiking (DH) (Via, 2012). DH facilitates the divergence of genes linked to genomic regions that are most involved in local adaptation, and may explain the evolution of clones in populations with extensive gene flow. In *E. faecium* clade A, DH may have contributed to the fixation of ecologically important genomic traits, like antibiotic resistance or genes linked to pathogenicity. Indeed, using a neutral model incorporating microepidemics and migration, which mimics a situation where ecological factors may limit transmission between subpopulations, showed that *E. faecium* hospital isolates with extensive genotype relatedness markedly deviated from this neutral model compared with other common nosocomial bacteria like *Staphylococcus aureus* and *Staphylococcus epidermidis*, indicating that these hospital isolates represent a subpopulation adapted to the hospital environment (Numminen et al., 2016).

The *E. faecalis* population analyzed in the studies described above included human commensal, farm animal and clinical isolates. It is an open question why this anthropogenic divergence, as observed in *E. faecium*, is not seen in *E. faecalis*. An explanation for this may reside in differences in population size of both species. Indeed, *E. faecalis* seems to be more widespread and abundant in the intestines of cattle, pigs, dogs, horses and poultry (Devriese et al., 1987, 1991, 1992a,b, 1994). *E. faecalis* has also been more frequently isolated from wild mammals, reptiles, birds, insects, and wild plants than *E. faecium* (Mundt, 1963a,b; Martin and Mundt, 1972). Finally, in healthy humans *E. faecalis* is also more frequently found than *E. faecium* (Tannock and Cook, 2002). Moreover, it seems that *E. faecalis* clones are maintained longer in the gut than *E. faecium* clones (Ruiz-Garbajosa et al., 2009). The higher population density of *E. faecalis* relative to *E. faecium*, in combination with its broader host-range, should provide *E. faecalis* with more opportunities for genetic exchange and diversification than *E. faecium*. High population densities also assure frequent transmission events, favoring gene flow among strains. This may act as a unifying force preventing the evolution of distinct clades in *E. faecalis*. Using the same neutral model described above for *E. faecium*, showed that the population structure of *E. faecalis* could be reflective of the evolutionary dynamics of a generalist organism which regularly experiences a high level of drift and gene flow between different host species (Numminen et al., 2016). Different strategies influence interactions of *E. faecalis* with populations of the same or of different species. A mechanism of substantial genetic exchange in *E. faecalis* has been postulated by Manson and co-workers, who described conjugative transfer of chromosomal fragments driven by integration of pheromone responsive conjugative plasmids carrying IS*256* insertions and recombination across IS*256* copies in the genome (Manson et al., 2010). The authors demonstrated that such an event could include transfer and recombination of up to 857 kb (∼ 25% of the genome) and show that essentially every chromosomal location can be transferred by this mechanism. Other strategies may include phages, mobile genetic elements and the generation of polymicrobial biofilms (Weigel et al., 2007; La Rosa et al., 2015, 2016; Gaca and Gilmore, 2016).

High-level genetic connectivity of strains between different niches and hosts might favor dissemination of adaptive elements (e.g., virulence and resistance genes) that are acquired by *E. faecalis* populations in one niche (e.g., animals raised for food production) and finally end up being selected for in another environment (e.g., the hospital). In fact, the panmictic structure of *E. faecalis* favors its behavior as a collective evolutionary individual, which has promoted the generalist lifestyle of *E. faecalis*. The high level of genetic connectivity of *E. faecalis* strains in different niches, in combination with a relative small genome size, is in accordance with the ‘Black Queen Hypothesis’. According to this hypothesis collective genes shared in a highly recombinogenic structure act as “common goods”, and may favor the loss of genes in a particular niche in individual strains, provided they are not lost entirely from the community (Morris et al., 2012). While there is evidence in favor of this hypothesis, there is still a distinct lack of studies using WGS to describe the evolution, genetic diversity and population structure of *E. faecalis* and these are urgently needed to validate or falsify the current view of *E. faecalis* as a generalist.

In conclusion, *E. faecium* and *E. faecalis* are widely distributed among humans, animals and the environment. The social behavior of enterococci, which includes the accretion of plasmids, phages and CTns (Werner et al., 2013; Clewell et al., 2014), but also the ability to maintain microbial communities (Barnes et al., 2012; Paganelli et al., 2013), has allowed them to rapidly acquire antibiotic resistance genes and genetic elements that increase their ability to colonize and infect patients. *E. faecium* and *E. faecalis* differ in their population structure. *E. faecium* has clearly defined sub-populations, one of which contains the majority of strains that are currently causing nosocomial infections. In contrast, *E. faecalis* is a generalist, with a highly panmictic population where genes involved in patient colonization or virulence are widely spread across niches.

## Author Contributions

Most of the writing for this manuscript was done by AG, WS, and RW, with MR contributing analyses for both figures and TC, FB, and JC contributing concepts and text in sections 3 and 4. All authors have contributed to and revised the manuscript prior to submission. In our opinion, all authors meet the requirement of authorship as outlined in the Frontiers author guidelines.

## Conflict of Interest Statement

The authors declare that the research was conducted in the absence of any commercial or financial relationships that could be construed as a potential conflict of interest.
